# Should We Be Using Upstream Beta-Blocker Therapy for Acute Myocardial Infarction?

**DOI:** 10.1007/s11886-021-01494-3

**Published:** 2021-05-07

**Authors:** Georgios Giannakopoulos, Stephane Noble

**Affiliations:** Cardiology Division, Department of Medicine, University Hospital of Geneva, Geneva, Switzerland

**Keywords:** Beta-blocker, Acute myocardial infarction, Cardiogenic shock

## Abstract

**Purpose of Review:**

Controversy exists whether beta-blockers should be given before primary percutaneous coronary intervention (PCI) or to defer their administration for up to 24 hours.

**Recent Findings:**

Animal studies, most of them conducted in the 1970s and 1980s, showed evidence that early beta-blocker administration may reduce infarct size. Subsequent human studies had mixed results on infarct size and survival. More specifically, in the current primary PCI era, only four studies evaluated the impact of early intravenous beta-blocker administration after acute myocardial infarction, only two of them before PCI. All studies agree that in hemodynamically stable patients, early intravenous beta-blocker administration is safe and protected against malignant arrhythmias. Nevertheless, results on infarct size and mortality are equivocal.

**Summary:**

Considering the heterogeneity of currently available data, further studies are still needed to assess the benefit of early injection of metoprolol in STEMI patients in a large double-blinded and randomized design versus placebo.

## Introduction

Ischemic heart disease remains the leading cause of death in the world despite important therapeutic progress over the last four decades. Acute myocardial infarction (MI) is a life-threatening manifestation of ischemic heart disease requiring emergency medical response. Mortality from acute MI has decreased in Western countries since the 1980s not only due to the introduction of prompt defibrillation, monitored units and early reperfusion therapies (initially thrombolysis in the 1980s and primary percutaneous coronary intervention (PCI) in the late 1990s) but also due to the routine use of modern antithrombotic therapies, statins, ACE inhibitors or angiotensin-receptor antagonists, and beta-blockers [1]. Nevertheless, in a report of the 2010/2011 data from the national registries of ST-segment elevation MI (STEMI) patients in 37 European countries, the in-hospital mortality rate remains between 4 and 12%, and a substantial number of STEMI patients (19 to 526 per 1,000,000 habitants) mostly in Eastern and Southern Europe did not receive any reperfusion therapy [2]. The *Stent For Life* initiative from the European Society of Cardiology is actively contributing to increase access to reperfusion therapies and more specifically to primary PCI. On the other hand, despite the studies in the 1980s showing the benefit of beta-blocker therapy in non-reperfused MI, there are still unanswered questions with respect to the use of beta-blockers in the context of acute MI, in particular the timing of administration and the optimal dose. In this review article, we will discuss the data over time and whether we should be using upstream beta-blocker therapy for acute MI.

### Beta-Blockers

The discovery of adrenergic receptors in the late 1940s [3] and the development of beta-adrenergic blockers in the mid-1960s, with propranolol being the first in clinical use [4], established the leading role of this medication class for all aspects of ischemic heart disease including angina relief, secondary prevention after MI, treatment of heart failure, and prevention of malignant arrhythmias.

Beta-blockers have a negative chronotropic effect decreasing heart rate, a negative inotropic effect that reduces myocyte contractility and a lowering effect on blood pressure. As a result, beta-blockers decrease myocardial oxygen consumption. Furthermore, they increase myocardial oxygen supply namely through prolonged diastolic filling time and decreased left ventricular filling pressure. In addition, beta-blockers increase the threshold of ventricular tachycardia and reduce ectopic automaticity, and by doing so they prevent sudden cardiac death [5].

## Animal Models

Researchers in the early 1970s studied the extent of ischemia induced by coronary artery ligation in anesthetized dogs. They found that myocardial infarct size, as measured by the sum of ST segment elevation on surface ECG and quantification of cardiac enzymes, was increased by positive chronotropic and inotropic agents such as isoproterenol and digital glycosides or rapid pacing and decreased when propranolol was administrated [6]. Remarkably, the cardioprotective effect of propranolol was maintained when heart rate was kept constant by electro-stimulation and when propranolol was infused as late as 3 h after ligation [6]. Nevertheless, Rasmussen and colleagues determined that delayed (> 3 h) propranolol administration after litigation of the circumflex artery in open-chested dogs halved the effectiveness of the treatment as quantified by histopathologic quantification of the necrotic heart muscle [7]. Other animal studies showed that beta-blockers reduce mitochondrial damage [8] and microvascular injury [9]. Nevertheless, the same results were not obtained with verapamil [10] implying that mechanisms specific to beta-blockers such as restoration of protective signaling pathways [11] and neutrophil-platelet interaction inhibition [12••] may play a role.

More recently in 2007, the effect of beta-blockers on infarct size was studied by Ibanez and colleagues in a swine model. The investigators measured the extend of the MI at cardiac magnetic resonance imaging and histopathology after 90 min of left anterior descending artery (LAD) balloon occlusion followed by reperfusion. Compared to placebo, intravenous metoprolol administrated 15 min after LAD occlusion reduced infarct size by a factor of 5 and significantly increased left ventricular ejection fraction (LVEF) at day 22 [13].

## Studies of Early Intravenous Beta-Blockers in Humans with Acute Myocardial Infarction

### Before the Reperfusion Era

A substantial number of randomized trials were performed before the adoption of fibrinolysis, albeit with mixed results; some showing reduction of infarct size and survival benefit [14] but others not [15, 16]. A meta-analysis published by Yusuf et al. in 1985 showed that early beta-blocker intravenous administration (typically followed by oral administration) resulted in a 20% reduction in cumulative cardiac enzyme release, better R-wave preservation and Q-wave reduction as well as significant reduction of ventricular ectopic beats and ventricular fibrillation. However, no significant benefit on early mortality could be detected [17].

### In Association with Reperfusion Using Thrombolysis Therapy

In a sub-study published in 1991 of the Thrombolysis in Myocardial Infarction II-B (TIMI II-B) trial, more than 1400 patients were randomized between immediate administration of intravenous metoprolol (15 mg given within a mean of 42 min after the beginning of thrombolytic therapy) and deferred administration of oral metoprolol initiated on day 6. The study found no difference in LVEF at discharge nor a decrease in death or reinfarction at 6 weeks and 1 year. However, the incidence of reinfarction and chest pain was lower in the intravenous group at day 6 [18].

The Clopidogrel and Metoprolol in Myocardial Infarction Trial (COMMIT) randomized more than 45,000 Chinese patients with acute MI (87% STEMI) to receive placebo versus 15 mg of intravenous metoprolol followed by 200 mg oral metoprolol daily. Only half of the study participants received fibrinolytic therapy and patients scheduled for angioplasty were excluded per protocol. There was no significant reduction in mortality in the beta-blocker arm at 28 days. Patients treated with high metoprolol doses had fewer reinfarctions and a lower incidence of ventricular fibrillation, but this was counterbalanced by a higher incidence of cardiogenic shock. Furthermore, there was a higher mortality rate in the beta-blocker group among Killip class III and hypotensive patients.

In addition, two meta-analyses including trials mostly from the pre-primary PCI era found that intravenous beta-blockers reduce mortality by a modest 8–13% with concomitant reduction of reinfarction and sudden death [19, 20].

### Current Primary PCI Era

Since 2012, four randomized trials investigated the effects of early intravenous beta-blockers in patients undergoing primary PCI for acute MI. In two (METOCARD-CNIC, 270 patients [21] and EARLY-BAMI, 683 patients [22]), intravenous metoprolol was given in the ambulance or at the PCI center before PCI was performed. In the other two trials, intravenous beta-blocker treatment was started shortly after PCI was performed (< 60 min for BEAT-AM, esmolol, 100 patients [23]) and directly after PCI in the study by Hanada et al. (landiolol, 96 patients [24]).

The METOCARD-CNIC trial (Effect of Metoprolol in Cardioprotection During an Acute Myocardial Infarction) randomized patients with anterior STEMI to receive up to 3 doses of 5 mg intravenous metoprolol (*n* = 139) or standard medical treatment (*n* = 131) before PCI. The median time interval between STEMI diagnosis and intravenous metoprolol administration was 10 min, and started within 6 h of chest pain onset. Killip class III and IV patients as well as patients with bradycardia, hypotension or atrioventricular block were excluded, and all patients received oral metoprolol within 24 h. Patients who received intravenous metoprolol had smaller infarct sizes at day 5 to 7 measured at cardiac MRI (25.6 g vs 32 g, *p* = 0.012) or estimated by the peak (2397 vs 3176 IU/L, *p* = 0.019) and area under the curve (49427 vs 62953 IU/L, *p* = 0.029) of creatinine kinase. At 6 months, patients in the intravenous beta-blocker group had higher MRI-measured LVEF (48.7% vs 45%, *p* = 0.025), lower incidence of severely reduced LVEF (11% vs 27%, *p* = 0.006) and fewer indications for ICD implantation (7% vs 20%, *p* = 0.012). At 2 years, patients in the treatment group also had fewer heart failure-related hospitalizations than patients in the control group [25].

In a post hoc analysis [26], patients were divided in two groups depending on the time interval between the first metoprolol injection and reperfusion, the median time interval of 53 min being used as the cut-off value. Patients in the long interval group (i.e., patients who received the first metoprolol injection at least 53 min before reperfusion) having thus a longer exposure to this medication had smaller infarct sizes (22.9g vs 28.1g, *p* = 0.06) and higher LVEF at day 5 (48.3% vs 43.9%; *p* = 0.019) compared to the short interval group, despite longer symptom-to-balloon time. Indeed, the authors calculated that every 10 min of “on board” metoprolol saved 1.1 g of myocardium and increased LVEF by 0.6%. This long exposure benefit was maintained at 6 months.

The EARLY-BAMI trial randomized 600 STEMI patients within 12 h of symptom onset to receive either intravenous metoprolol (5 mg at recruitment and 5 mg before PCI) or placebo before primary PCI. In contrast to the METOCARD-CNIC trial, the EARLY-BAMI trial included all STEMI localizations and a substantial number of patients (18.8%) already on beta-blockers. Similar to the METOCARD-CNIC trial, Killip III and IV patients, patients with heart rate < 60 beats/min, with blood pressure < 100 mmHg, or with second-or third-degree atrioventricular block were excluded. Infarct size measured at cardiac MRI and estimated by CK peak and area under the curve did not differ between the two groups. LVEF was 51% in the metoprolol group and 51.6% in the placebo group (*p* = 0.68). Of note, there was a borderline reduction in malignant arrhythmias (*p* = 0.050) without any increase of symptomatic bradycardia, hypotension or cardiogenic shock in the beta-blocker group.

It is worth including in the discussion the BEAT-AMI trial [23] and the study by Hanada et al. [24] despite the fact that in these trials, the beta-blockers were administrated after the primary PCI. The BEAT-AMI trial randomized 100 STEMI patients in Killip I and II classes to placebo versus continuous esmolol infusion started within 60 min post primary PCI. Cardiac enzymes and NT-proBNP were significantly lower in the treatment group, and no patient in the esmolol group presented cardiogenic shock (3 in the placebo group). Hanada et al. administrated continuous i.v. landiolol, an ultra-short-acting cardio-selective beta-blocker, to half of their study’s population (96 patients). Infusion started immediately after primary PCI and was continued for 24 h. There were no major adverse events and an improvement in LVEF between 2 weeks and 6 months in the landiolol group, but not in the control group.

Finally, in 2019 a patient-pooled meta-analysis of the four abovementioned trials including a total of 1150 patients found that early intravenous beta-blocker administration does not increase a composite safety endpoint of cardiogenic shock, symptomatic bradycardia or hypotension in Killip I and II patients, but does improve LVEF at 6 months, and does not decrease all-cause mortality at 1 year (Table 1) [27••]. Indeed, for the physicians who are reluctant to administer early intravenous beta-blockers because of the risk of adverse events (i.e., cardiogenic shock, hypotension or bradycardia), they can be reassured by this meta-analysis that early intravenous beta-blocker administration is safe in selected STEMI patients undergoing primary PCI.

**Table 1:** Table 1

Study names	Number of patients	Beta-blocker	Randomized versus	Inclusion criteria	Exclusion criteria	Results
2013 METOCARD-CNIC^21,25^	270 Multicenter Spain	IV metoprolol before PCI up to 3 doses (15 mg) 99% -> 5 mg 82% -> 10 mg 67% -> 15 mg	Routine care Single-blind	Anterior STEMI within 6 h for reperfusion 4.5h for inclusion	Killip 3-4 SBP<120 mmHg HR < 60 bpm PR>240 msec AV block II-III Already on BB	No excess adverse events at day 5-7 (MRI + peak/area under the CK curve) Reduction of MI size at 6 months Higher LVEF, less LVEF<35%, less ICD at 2 years less HF admission
2016 EARLY-BAMI^22^	683 Multicenter Spain and Netherlands	IV metoprolol before PCI 5 mg in ambulance 5 mg just before PCI (81% received the second bolus)	Placebo Double-blind	STEMI within 12h	Killip 3-4 HR < 60 bpm SBP<90 mmHg AV block II-III Asthma Pacemaker Previous MI	BB reduced malignant arrhythmias in acute phase Equal adverse events At 30 days (MRI+CK) No difference in MI size and LVEF
2016 BEAT-AM^23^	100 single center Germany	IV esmolol post PCI	Placebo Single-blind	STEMI within 6h	Killip 3-4 HR < 60 bpm mBP<65 mmHg	BB significantly decreased troponin T, CK, CK-MB and NT-pro-BNP No cardiogenic shock in the esmolol group (3 in the placebo group)
2012 Hanada et al^24^	96 single center Japan	IV landiolol post PCI perfusion for 24h	Routine care Non-blinded	STEMI within 12h	Killip 3-4 HR < 50 bpm SBP<90 mmHg Bronchospasm AV block II-III	No differences in cardiac event in the acute phase At 6 months LVEF higher in BB group LVEDVI increased in the control group

AV: atrio-ventricular, BB: beta blocker, HF: heart failure, HR: heart rate, ICD: intra-cardiac defibrillator, IV: intravenous, LVEDVI: left ventricular end-diastolic volume index, LVEF: left ventricular ejection fraction, BP: mean blood pressure, MI: myocardial infarction, MRI: magnetic resonance imaging, PCI: percutaneous coronary intervention, SBP: systolic blood pressure

## What Do the Current Guidelines Say on Upstream Intravenous Beta-Blocker Administration?

### European Guidelines

In the latest guidelines for the management of STEMI, the European Society of Cardiology downgraded the use of beta-blockers. Indeed, in 2015 early administration of beta-blockers for ongoing ischemia was a Class I, level of evidence (LOE) B. In 2017, they gave a Class IIa, LOE A for intravenous beta-blocker administration at the time of presentation for patients undergoing primary PCI (without signs of acute heart failure and with systolic blood pressure (SBP) > 120 mmHg). Importantly, it is emphasized that intravenous beta-blockers are harmful for patients with hypotension, acute heart failure, atrioventricular block or severe bradycardia (Class III, LOE B) [28].

Similarly, routine in-hospital and chronic use of beta-blockers was downgraded from a Class I LOE B in 2015 to a Class IIa, LOE B in 2017.

The 2020 European guidelines for the management of non-STEMI recommend the early initiation of beta-blockers in association with nitrates for symptom relief (Class I and LOE C). Based on an observational study by Kontos et al. [29] including more than 21,000 NSTEMI patients, early use of beta-blockers should be avoided in patients at risk of cardiogenic shock (i.e., age older than 70 years, heart rate > 110 bpm or SBP < 120 mmHg) especially when the LVEF is unknown [30]. Indeed, in this study patients at risk of cardiogenic shock who received beta-blockers very early in the emergency department compared to patients with later administration of beta-blockers, but within 24 h of hospital admission, experienced significantly more death and cardiogenic shock.

### American Guidelines

Similarly, current ACC/AHA guidelines from 2013 for the management of STEMI recommended the intravenous administration of beta-blockers to patients at the time of STEMI if they are hypertensive or present ongoing ischemia (Class IIa, LOE B) [31].

The 2014 ACC/AHA non-STEMI guidelines focus on the potential harm of intravenous beta-blockers in patients with risk factors for cardiogenic shock, giving thus a Class III, LOE B recommendation for this population [32].

Both STEMI and NSTEMI guidelines strongly recommend (Class I, LOE B) oral beta-blocker therapy to be initiated within the first 24 h in patients without signs of heart failure, evidence of low cardiac output, increased risk of cardiogenic shock or other contraindications to beta-blockers (PR interval > 0.24 sec, second- or third-degree heart block without a cardiac pacemaker, active asthma, or reactive airway disease).

Finally, the usual recommendation is that beta-blockers should not be administered in patients with possible coronary spasm or cocaine use, since beta-blockers might favor spasm by leaving alpha-mediated vasoconstriction unopposed by beta-mediated vasodilatation.

## Perspective

Animal studies showed that the timing of administration plays a role on the benefit of beta-blockers. In clinical practice, the COMMIT trial (high metoprolol dose, fewer reinfarction, and incidence of ventricular fibrillation, but a higher rate of cardiogenic shock) demonstrated that the dosage and the selection of patients matter. Indeed, there was higher mortality rate in the beta-blocker group among Killip class III and hypotensive patients.

The major studies in the primary PCI era were included in a meta-analysis of 1150 patients confirming the safety of early beta-blocker injection in selected STEMI patients with no increased occurrence of cardiogenic shock, hypotension, or bradycardia. However, there was no difference in the main outcome of death or MI at 1 year after early beta-blocker injection. Importantly, between the two largest trials of this meta-analysis (representing 83% of the meta-analysis cohort), METOCARD-CNIC [21] (270 patients) and EARLY-BAMI [22] (683 patients), there were conflicting results with respect to the reduction of MI size. Indeed, the METOCARD-CNIC trial showed a significant reduction in MI size. The population of this study had larger MI, MI only localized on the anterior wall, included within only 6 h of symptom onset and receiving 15 mg of intravenous metoprolol. However, it was not blinded nor a placebo-controlled trial [21]. On the contrary, EARLY-BAMI trial included patients with any location of MI, within 12 h of symptom onset, and the intravenous metoprolol administered was only 10 mg and closer to the PCI, therefore with less “on board” time. However, it was a blinded placebo-controlled trial [22].

Therefore, considering the safety of early beta-blocker injection and the differences in the 4 trials included in the meta-analysis, additional studies are needed to answer when beta-blockers are really needed, namely, in the ambulance, at the emergency department or post primary PCI.

Reopening the vessel is essential, but beta-blockers have the potential to decrease the infarct size. Data are stronger for metoprolol than for the other beta-blockers. A recent study suggested that neutrophil stunning induced by metoprolol might be a potential explanation for the reduced MI size, knowing that according to both animal and human studies, that MI size is related to a pro-inflammatory response occurring during and post-MI and exacerbated by reperfusion [12, 33]. This neutrophil stunning by metoprolol does not seem to be shared by other beta-blockers [34••].

Considering the data heterogeneity, as stated by the authors of the meta-analysis [27••], further randomized studies should assess the benefit of early injection of 15 mg of metoprolol in STEMI patients (with subgroup analysis of anterior and non-anterior MI, small and large MI) in a large double-blinded and randomized design versus placebo. However, considering the lack of return on investment, there is a low likelihood that the pharmaceutical industry will fund such a trial. Therefore, medical societies or public funds or foundations should finance such a project.

Finally, beyond the acute phase, there are still a few unanswered questions, in particular the optimal dose and duration as well as the benefit of beta-blockers in patients with normal or mildly depressed LVEF. Given the frequent side effects and discontinuation of the treatment, as well as the lack of data in the primary PCI era, several randomized controlled trials are currently investigating these questions such as AbYSS (Beta-Blocker Interruption After Uncomplicated Myocardial Infarction, NCT03498066), REDUCE-SWEDEHEART (Evaluation of Decreased Usage of Betablockers After Myocardial Infarction in the SWEDEHEART Registry, NCT 03278509), and REBOOT-CNIC (TREatment With Beta- blockers After myOcardial Infarction withOut Reduced Ejection fraction, NCT03596385) [35].

## Conclusion

Upstream administration of intravenous beta-blockers, and in particular metoprolol, before primary PCI is safe and supported by the clinical guidelines in selected patients with Killip I and II acute MI, SBP > 100 mmHg, and heart rate > 60 beats/min, whereas it should be avoided in patients with cardiogenic shock. The treatment in the acute phase decreases the incidence of malignant arrhythmias and may improve LVEF at 6 months. Furthermore, there is some evidence that it may decrease the MI size especially when given early, preferably in the ambulance, allowing longer “on board” time. However, considering the heterogeneity of currently available data, further randomized studies are still needed to make definitive conclusions.
